# Supplementary material for: Long-Distance Electrical and Calcium Signals Evoked by Hydrogen Peroxide in Physcomitrella
Source: Plant Cell Physiol. 2023 May 26;64(8):880–92. doi: 10.1093/pcp/pcad051 (PMC10434737; doi:10.1093/pcp/pcad051)
Supplement: pcad051_Supp [file pcad051_supp.zip › suppl_data/pcp-2023-e-00099-File016.docx]

**Supplementary Video Legends**

**Supplementary Video 1**. Comparison of H_2_O_2_-evoked membrane potential changes recorded in the protonema cell after stimulation of different regions of the plant. Membrane potential changes were recorded in the same cell during the experiment which initially started from microinjection of 0.5 mM H_2_O_2_ stained with 1 mM methyl blue into the chain of protonema cells at a distance from the tested cell with the inserted microelectrode; next, the microinjection was directed to the tested cell and finally to the basal part of the plant. Membrane potential changes recorded during the stimulation are visible in the right panel. The video recording is four times faster than in real time.

**Supplementary Video 2**. Changes in H_2_O_2_-evoked membrane potential transmitted from cell-to-cell along the chain of protonema cells. The method of the experiment was the same as the initial part of experiment presented in Supplementary Video 1, where microinjection of 0.5 mM H_2_O_2_ stained with 1 mM methyl blue was applied to the chain of protonema cells at a distance from the tested cell.

**Supplementary Video 3.** Comparison of H_2_O_2_-evoked membrane potential changes recorded in the protonema cell from Pp*glr1*^KO^ mutant after stimulation of different regions of the plant. The method of the experiment was the same as in Supplementary Video 1.

**Supplementary Video 4**. Measurements of fluorescence of calcium signals recorded in the GCaMP3 mutants of Physcomitrella. The calcium signals were evoked by microinjection of 0.5 mM hydrogen peroxide stained with 0.025 µM fluorescein into the base of the gametophyte. The two fragments of protonema marked by the rectangles were chosen for the analysis of the direction and velocity of calcium signals. The video recording is forty times faster than in real time.

**Supplementary Video 5.** Different types of propagation of calcium signals recorded in the GCaMP3 mutants of Physcomitrella. The method of experiment was the same as in Supplementary Video 4. In the analyzed fragments of protonema, three different types of calcium signal propagation were observed - from the place of stimulation (marked by red ellipse), in opposite direction (blue ellipse) and diminishing with the distance (white ellipse).

**Supplementary Video 6**. Effects of 0.5 mM and 5 mM H_2_O_2_ application on calcium response recorded in leaf cells from GCaMP3 mutants of Physcomitrella.

**Supplementary Video 7.** Calcium signals recorded in leaves of GCaMP3 mutants of Physcomitrella after application of 0.5 mM H_2_O_2_ on the basal part of the gametophyte.
